# Long-Term Clinical Outcome of Low-Temperature Plasma Ablation Treatment for Recurrent Corneal Erosions

**DOI:** 10.3390/jcm11216280

**Published:** 2022-10-25

**Authors:** Yu Zhang, Lin Lin, Yirui Zhu, Shuo Yang, Xiaodan Huang

**Affiliations:** Eye Center, The Second Hospital, School of Medicine, Zhejiang University, Hangzhou 310000, China

**Keywords:** low-temperature plasma ablation, recurrent corneal erosions, microcysts, corneal in vivo confocal microscopy, corneal topography

## Abstract

Purpose: To observe the therapeutic effect of low-temperature plasma ablation in treating patients with recurrent corneal erosions (RCEs). Materials and Methods: From 2020 to 2022, 35 participants with unilateral RCEs voluntarily enrolled. Here, 35 eyes of 35 patients were treated with low-temperature plasma ablation (coblation). All surgeries were performed by the same doctor (X.H.). The coblation went back and forth over the entire erosion area around five times for about five minutes. After the operation, patients were scheduled for follow-up visits at the outpatient clinic after 1 month, 3 months, 6 months, and 12 months, and at the end of the trial. During every visit, each patient underwent an evaluation of their ocular symptoms utilizing the following: pain score, intraocular pressure, slit lamp biomicroscopic examination, dry eye analysis, corneal topography, and corneal in vivo confocal microscopy (IVCM). Results: The mean follow-up time was 12.4 ± 6.1 months, ranging from 6 to 29 months. Of the 35 patients who had low-temperature plasma ablation, 32 eyes (91.43%) were completely symptom-free and three (8.57%) eyes had repeated episodes of recurrent corneal erosions after the surgery, at 3, 10, and 12 months, respectively. The typical RCE morphologic abnormalities observed in IVCM included the deformation and relaxation of the corneal epithelium, disorganized stromal fibers, reduced nerve fiber density, and disordered organization. After low-temperature plasma ablation, the loose cysts disappeared, and the epithelium became solid and tight. There was no obvious difference in the intraocular pressure (*p* = 0.090) or corneal astigmatism (*p* = 0.175) before and after treatment. The mean pain score decreased significantly, with a preoperative score of 7.7 ± 2.4 and postoperative score of 1.1 ± 1.8 (*p* < 0.001). The mean corneal thickness decreased from 562.6 ± 42.2 mm to 549.6 ± 26.9 mm (*p* = 0.031). The mean non-invasive keratograph tear meniscus height (NIKTMH) decreased from 0.210 ± 0.054 mm to 0.208 ± 0.045 mm (*p* = 0.001), and the mean TBUT decreased from 6.191 ± 2.811 s to 5.815 ± 2.802 s (*p* < 0.001), which manifested as a slight worsening of dry eyes. In one case, the patient’s corneal astigmatism became more severe, which may be related to the high instantaneous energy when the operation did not flush in a timely manner, and it was recovered after 6 months. **Conclusions**: Low-temperature plasma ablation is an effective and safe procedure to treat patients with recurrent corneal erosions.

## 1. Introduction

Recurrent corneal erosion (RCE) is a common chronic relapsing disease. Episodes are characterized by the sudden onset of ocular pain, lacrimation and photophobia, which often occur upon waking [1]. Rapid eye movement (REM) or even a momentary opening of the eyes during sleep may induce acute pain, which rapidly wakes patients [2]. The length of these episodes can vary, ranging from minutes to hours to days, and they might occur frequently or merge into a continuous period of suffering [1]. According to the literature, trauma and epithelial basement membrane degradation (EBMD) are the two most typical causes [3,4,5,6,7]. Fingernail injury was shown to be the most prevalent mechanism in a subgroup study of the different causes of RCEs, followed by exposure to vegetable debris [5]. In 90% of cases, corneal erosion is unilateral [8] and mostly affects the inferior paracentral cornea [9]. The histopathology of RCEs is epithelial microcysts [10,11] and the displacement or duplication of the basement membrane into the epithelium [12,13,14]. Gelatinase, which comprises matrix metalloproteinase-9 (MMP-9) and matrix metalloproteinase-2 (MMP-2), degrades type IV collagen, type VII collagen and laminin, which are all essential components of the basement membrane. Increased MMP-9 and MMP-2 expression has been linked to the pathophysiology of RCEs because it can result in basement membrane breakdown and impaired epithelial basement membrane adhesion [14].

Conservative treatments of RCEs includes topical lubricants [15], topical hypertonic saline [15], topical steroids [15], autologous serum [16], bandage contact lenses [17], metalloproteinase inhibitors doxycycline and corticosteroids [18,19] and so on. However, these interventions offer patients only short-term improvements. Surgery is usually considered for patients who do not respond well to conservative therapy. Various surgical therapy options have been documented for RCEs, including an anterior stromal puncture (ASP) with a needle [20] or with a neodymium:yttrium-aluminum-garnet laser (Nd:YAG) [21], alcohol delamination (ALD) [22], diamond burr superficial keratectomy (DBSK) [23] and phototherapeutic keratectomy (PTK) [24].

Low-temperature plasma ablation (coblation), also known as cold-ablation, plasma-mediated ablation or bipolar radiofrequency ablation [25], is a novel technology in the field of ophthalmology. By transmitting radiofrequency energy through a conducting medium (usually normal saline), coblation creates a plasma field. At a relatively low temperature, a high quantity of plasma transmits energy to the tissue, resulting in the rupture of organic bonds and the breakdown of molecular structures. This technique can prevent heat damage to the surrounding tissues, which is beneficial for minimizing post-operative discomfort, improving tissue healing, lowering the risk of infection and reducing the possibility of post-operative hemorrhaging [26]. Coblation has been applied in otorhinolaryngology [26,27,28,29], urology [30] and orthopedic surgery [27], but it is rarely reported in ophthalmology.

The main purpose of the current study was to apply low-temperature plasma ablation surgery to treat RCEs, analyze its safety and efficacy by evaluating clinical indicators before and after treatment, evaluate its clinical application value and significance, and provide a reference for clinical treatment.

## 2. Materials and Methods

### 2.1. Study Design

We conducted this prospective, single-center, single-masked clinical trial from 2020 to 2022 at the outpatient clinics of the Eye Center, the Second Affiliated Hospital, Zhejiang University School of Medicine, in adherence to the Declaration of Helsinki. The study was authorized by the Ethics Committee of the Second Affiliated Hospital at Zhejiang University School of Medicine (20210264). All participants freely signed an informed consent form prior to taking part in the study. A total of 35 patients with RCEs were enrolled. Patients were scheduled for follow-up visits preoperatively, 1, 3, 6 and 12 months postoperatively, and lastly before the end of our study. The main assessments included a subjective symptom questionnaire, intraocular pressure, slit lamp biometric microscopy, corneal fluorescein staining, dry eye examination, in vivo confocal corneal microscopy (IVCM, HRT-3, Heidelberg Engineering GmbH, Heidelberg, Germany) [31] and corneal topography. The main outcomes included recurrence after treatment, pain scores (visual analogue scale, VAS), tear film breakup time (TBUT), non-invasive keratograph tear meniscus height (NIKTMH), corneal nerve fiber length (CNFL), corneal astigmatism, corneal thickness, IVCM image changes and postoperative complications. No other adverse reactions were observed during the follow-up.

### 2.2. Inclusion Criteria

The inclusion criteria were as follows: patients who had a typical history of eye pain, watering and increased sensitivity to light on waking, obvious signs on the slit lamp examination, and no response to conservative treatments for over 3 months, including regular topical lubricants and therapeutic contact lenses [4].

### 2.3. Exclusion Criteria

The exclusion criteria were as follows: participants with any corneal dystrophy other than epithelial basement membrane dystrophy, as well as systemic disorders known to be linked to recurrent corneal erosions, such as epidermolysis bullosa [4].

### 2.4. Low Temperature Plasma Ablation Surgery

All surgeries were performed by the same doctor (X.H.). The patients were first given an ocular topical anesthetic (Proparacaine Hydrochloride Eye Drops, Alcon, United States). An eyelid opener was used to open the eyelids. After this, the loose corneal epithelium was ripped using forceps. An ophthalmic plasma surgical system (PLA-700, MECHAN, Chengdu, China) was used to perform the round-trip reciprocating ablation of local wounds with an MC409 low-temperature plasma head mediated with normal saline. The energy used for ablation was 40–45 eV. The coblation went back and forth around five times over the entire area where the epithelium was ripped until the operation was completed. The total coblation time is usually about 5 min. After surgery, the patients had to wear a bandage contact lens (BCL) for a month, as well as use local fluorometholone eye drops (Shiga Factory of Santen Pharmaceutical Co. Ltd., Shiga, Japan) three times a day for 14 days and sodium hyaluronate eye drops (HYCOSAN, EUSANGmbH, Saarbrücken, Germany) three times a day for 30 days.

### 2.5. Statistics

All statistical analyses were conducted using version 26 of the IBM Statistical Package for the Social Sciences (SPSS) software package for Windows (IBM, Chicago, IL, USA). The descriptive statistics are summarized as the mean± SD. A Kolmogorov-Smirnov test was used to assess the normality of the continuous variables. Paired *t*-tests were used to assess the differences before and after the surgeries. The analysis was double-sided, and a *p* < 0.05 was considered statistically significant.

## 3. Results

### 3.1. Demographics and Medical Status

**Figure 1:**
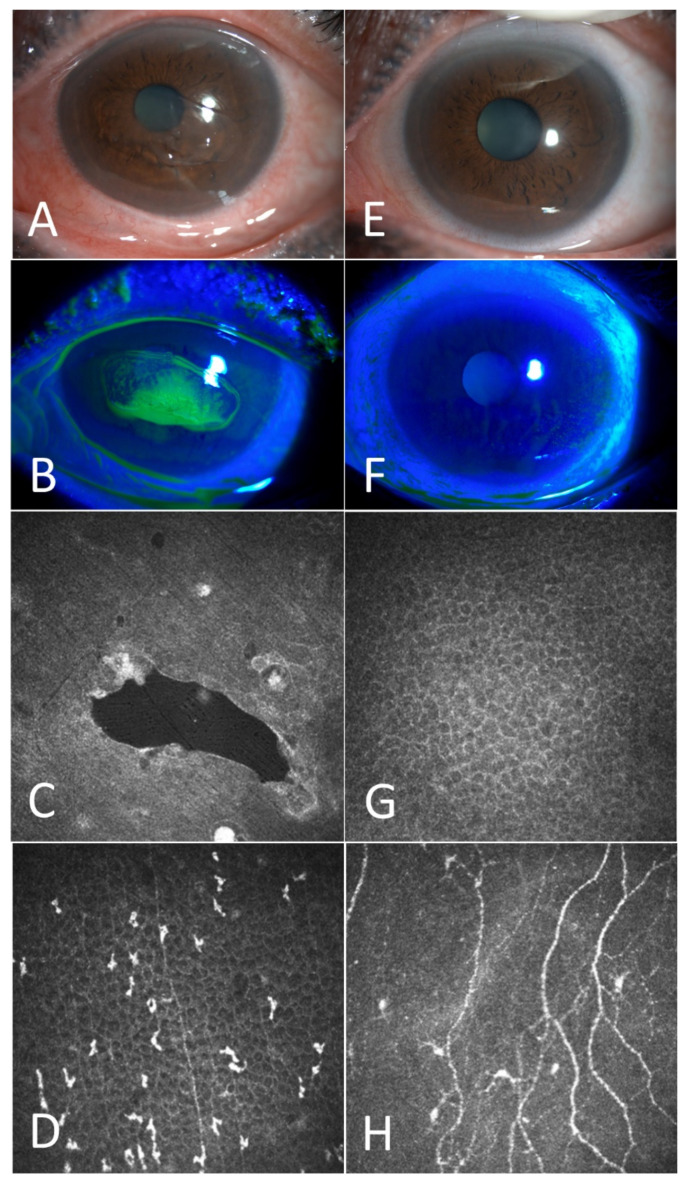
A case example before and 3 months after the coblation. Before the surgery: The corneal epithelium showed obvious detachment and corneal edema (**A**); Under cobalt blue light, fluorescein accumulates in the corneal loose area (**B**); Confocal microscopy revealed obvious cysts (**C**); corneal nerve reduction and increased activation of Langerhans cells (**D**). Three months after the surgery: Corneal edema decreased and corneal loose area disappeared (**E**,**F**); Confocal microscopy revealed that corneal epithelium became intact (**G**); bright reflective particles decreased and corneal nerves increased (**H**).

**Figure 2:**
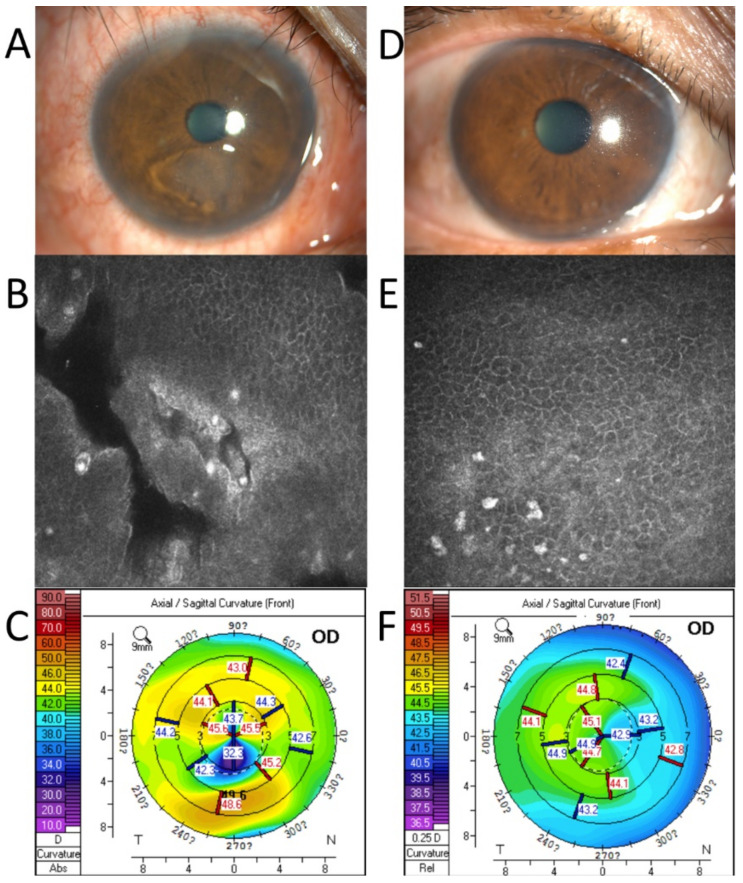
A case example before and 1 month after the coblation. Before the surgery: The corneal epithelium showed obvious detachment under the pupil (**A**); Confocal microscopy revealed obvious gaps (**B**); The corneal topography showed marked corneal astigmatism (**C**). One month after the surgery, the cornea healed (**D**); Confocal microscopy showed an intact corneal epithelium (**E**); Corneal astigmatism decreased significantly (**F**).

As can be seen in Table 1, 35 participants were enrolled, comprising 16 males and 19 females. The mean age was 48.9 ± 10.9 years, ranging from 25 to 67 years. The mean follow-up time was 12.4 ± 6.1 months, ranging from 6 to 29 months. The etiology was trauma in 16 patients (45.7%) and others, including epithelial basement membrane dystrophy (EBMD) and no specific reason in 19 patients (54.3%). The duration of RCEs is about 3 months (six patients, 17.1%), about 6 months (10 patients, 28.6%), about 12 months (16 patients, 45.7%) and about 24 months (three patients, 8.6%). Additionally, the mean duration is 9.8 ± 5.7 (3–24) months. The frequency of RCE recurrence is once a month (20 patients, 57.1%), once every two months (six patients, 17.1%), once every three months (five patients, 14.3%) and once every six months (four patients, 11.4%). Preoperative eye pain was assessed using the VAS scale: a score of 3 in two patients (5.7%), 5 in five patients (14.3%), 6 in nine patients (25.7%), 7 in three patients (8.6%), 8 in nine patients (25.7%), 9 in one patient (2.9%), 10 in twelve patients (34.3%). Additionally, the mean eye pain score is 7.7 ± 2.4 [3,4,5,6,7,8,9,10]. All patients had histories of eye pain, watering, and increased sensitivity to light on waking as well as typical RCE manifestations under the slit lamp examination. Under slit lamp white light, corneal epithelial detachments, cornea edema, and conjunctival injection were observed (Figure 1A and Figure 2A). After the instillation of fluorescein dye in the tear film (blue cobalt light with a yellow filter), fluorescein accumulated between the bullae (Figure 1B).

### 3.2. The Main Outcomes of Coblation

All operations were successful 1 month after the coblation surgeries. The definition of success in this treatment was defined as: on the slit lamp examination, the corneal epithelium was intact with no loose areas, and the patient’s subjective symptoms were alleviated. Recurrences of RCEs occurred in three of the 35 patients (8.6%), at 3, 10, and 12 months, respectively. None of the other patients had recurring issues during the follow-up period. As depicted in Table 2, the preoperative mean intraocular pressure was 15.5 ± 3.3 and postoperatively it was 16.9 ± 3.3. The preoperative mean corneal astigmatism was 1.5 ± 1.6 D, and the postoperative mean corneal astigmatism was 0.9 ± 1.1 D. The mean intraocular pressure and corneal astigmatism showed no significant change before and after the surgery, with *p* values of 0.090 and 0.175, respectively. The mean pain score decreased significantly, with a preoperative score of 7.7 ± 2.4, and a postoperative score of 1.1 ± 1.8 (*p* < 0.001). The mean corneal thickness decreased from 562.6 ± 42.2 mm to 549.6 ± 26.9 mm (*p* = 0.031). The mean NIKTMH decreased from 0.210 ± 0.054 mm to 0.208 ± 0.045 mm (*p* = 0.001). The mean TBUT decreased from 6.191 ± 2.811 s to 5.815 ± 2.802 s (*p* < 0.001). Additionally, the CNFL decreased from 10.106 ± 4.657 mm/mm^2^ to 6.928 ± 6.620 mm/mm^2^ (*p* = 0.028). In one patient, corneal astigmatism was aggravated from 1.5D to 8.3D, which may be related to the high transient energy because of the delayed flushing during the operation, and it recovered after 6 months. No other adverse effect was found in this study.

### 3.3. The Outcomes of In Vivo Confocal Microscopy

**Figure 3:**
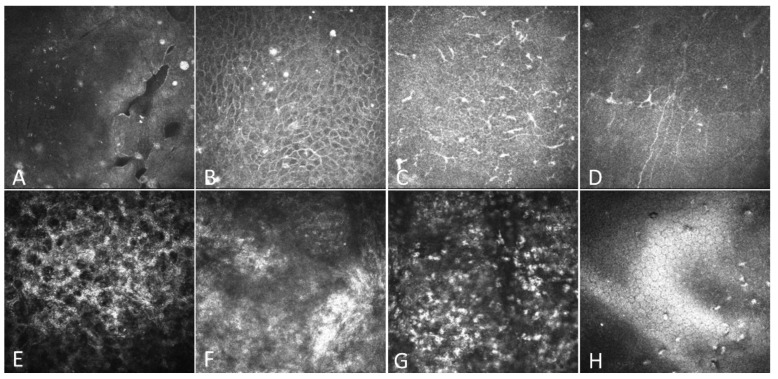
IVCM findings of the cornea in patients with RCEs. All images were obtained using the HRT-3-RCM system with a field of 400 µm × 400 µm. (**A**) Microcysts and gaps due to epithelial defects. (**B**) Elongated superficial cells and brightly reflective granular structures. (**C**) Increased activation of Langerhans cells. (**D**) Decreased corneal subbasal nerves. (**E**) Activated keratocytes in the shallow stroma. (**F**) Disorganized stroma fibers. (**G**) Infiltrated cells in the middle stroma. (**H**) Deposit on the endothelium.

As shown in Figure 3, all RCE patients showed irregular arrangements of epithelial cells (Figure 3B), bright reflective deposits in the epithelium (Figure 3B) and Bowman’s layer (Figure 3C), enlarged basal epithelial cells (Figure 3B), reduced corneal sub-basal nerves (Figure 3D), activation of shallow stroma (Figure 3E), and disorganized stroma fibers (Figure 3F). Thirty-one patients (88.6%) had microcysts or gaps in the epithelial cell layers (Figure 3A). Twelve patients (34.3%) had infiltrated cells, likely neutrophils, in the mid-stroma (Figure 3G). Six patients (17.1%) had bright precipitates on the endothelium (Figure 3H). After the operation, the epithelium completely healed, the cysts and gaps disappeared, and the bright reflective particles in the epithelium decreased significantly.

### 3.4. Case Examples

Patient 1. A 66-year-old woman with RCEs for 2 years received coblation surgery at our clinic. The preoperative examination revealed a large area of loose corneal epithelium in front of the pupil (Figure 1A). Fluorescein had accumulated in the loose area under cobalt blue light (Figure 1B). The IVCM examination revealed significant vesicles (Figure 1C) in the corneal epithelium, the accumulation of Langerhans cells (Figure 1D) in the Bowman’s layer and a significant decrease in corneal nerves (Figure 1D). Three months after the surgery, under the slit lamp, the cornea was clear and transparent and corneal edema and conjunctival congestion had decreased (Figure 1E,F). The IVCM examination showed the healing of the corneal epithelium (Figure 1G), a decrease in bright reflective particles in the Bowman’s layer and an increase in corneal nerves (Figure 1H).

Patient 2. A 62-year-old man with two months of trauma. The preoperative examination revealed a large area of loose corneal epithelium (Figure 2A,B). The IVCM revealed a clearly loose corneal epithelium. Corneal topography showed 7.5 D astigmatism (Figure 2C). One month after the surgery, the cornea had healed completely (Figure 2D), the loose area had disappeared (Figure 2E), and the corneal astigmatism had decreased to 0.9 D (Figure 2F).

## 4. Discussion

The present study has described the long-term outcomes of low-temperature plasma ablation treatment for recurrent corneal erosions. A total of 100% of the 35 treated eyes with visually evident epithelial erosions recovered one month following surgery. Both the mean intraocular pressure and the mean corneal astigmatism remained steady, demonstrating the safety of this procedure. The considerable decrease in the mean pain score demonstrated the effectiveness of the treatment. As a result, low-temperature plasma ablation is a safe and efficient operation for the treatment of recurrent corneal erosions.

RCE is a complicated disease which afflicts patients all over the world. Conservative treatment can be beneficial in the early stages of RCE and with minor visual axis disorders, such as map modifications that slightly impair vision. Medical treatments include lubricating ointments, hypertonic NaCl drops and hypertonic ointments [4]. Erythromycin or tetracycline ophthalmic ointments can reduce matrix metalloproteinase-9, which could contribute to the recurrent erosions [32]. Collagen shields and bandage contact lenses (BCL) [33] have also been used for recurrent erosions, but it is preferable not to use BCL for an extended period because of the potential problem of bacterial ulceration. However, in many cases, conservative treatments do not solve the problem, so surgery is needed. According to reports, available surgeries include an anterior stromal puncture (ASP) with a needle [20] or with a neodymium:yttrium–aluminum–garnet laser (Nd:YAG) [21], alcohol delamination (ALD) [22], diamond burr superficial keratectomy (DBSK) [23] and phototherapeutic keratectomy (PTK) [24]. However, an ASP can leave a puncture scar and should not be used in the pupil area. DBSK can lead to an obvious astigmatism according to Wong et al. [34]. Hyperopic shifts [35] and self-resolving subepithelial haze [22] have been reported with PTK.

Low-temperature plasma ablation is a new technique widely used in otorhinolaryngology [26,27,28,29]; however, its application in ophthalmology is rare. Huang H. et al., concluded that low-temperature plasma ablation is effective and safe for treating pterygiums. It reduces the amount of time needed for surgery, reduces inflammation and improves the graft stability without raising the risk of complications [36]. The results of Wahaj Saleem ‘s experiment show that the treatment enhances the rate of scratch wound closure in a human corneal epithelial (HCE) cell line and considerably reduces the incidence of mucopurulent corneal ulcers and the severity of edema (swelling) in *P. aeruginosa*-infected rabbit corneas [37]. A thin layer of plasma vapor about ≤50 μm thick is formed around the electrode after using low-temperature plasma ablation through a saline medium. This thin layer of particles is not driven by thermal energy, and has enough energy to smash organic molecular chains in the tissue, thus allowing molecules to separate from the cells of the target tissue. During the operation, a large amount of plasma transfers energy to the soft tissue at a relatively low temperature, resulting in the rupture of organic bonds, the destruction of molecular structures and, thus, tissue ablation [38]. The equipment we used, a MECHAN plasma knife, has an improved technique. After modification, the MECHAN plasma knife can allow low-temperature plasma in the electric field of 40–45 eV to perform rapid movement to generate kinetic energy, which can cause tissue decomposition to achieve the role of cutting. At the same time, with a cold saline injection, the temperature of the knife head is well controlled, which is generally stable at 25–28 ℃, and the probability of tissue burns becomes very low. The ablation depth can be controlled at about 50μm, and the ablation only affects the anterior corneal stromal layer, so it is safe and suitable for the treatment of eye diseases. When ablating conjunctiva and sclera, the energy is controlled at 40~50 eV, as set by the machine, and when ablating the cornea, the energy is controlled at 40~45 eV, which can completely ablate new biological tissues with almost no bleeding. In the case of a bleeding point, hemostasis can be stopped quickly with a hemostasis function of 50 eV, whereas the traditional method requires cauterization to stop bleeding. Several Chinese ophthalmic journals have published reports on the safety and efficacy of the MECHAN plasma system for the treatment of ocular surface diseases. Tan QW et al., concluded that MECHAN low-temperature plasma ablation using an MC-409 combined with drug therapy could effectively control infections of and promote healing for infected corneal ulcers, with no obvious complications observed [39]. Luo SR et al., demonstrated that low-temperature plasma ablation with an MC-409 was effective for the treatment of fungal corneal ulcers and has obvious therapeutic outcomes [40]. Cai et al., observed that the treatment of papilloma, conjunctival granulation hyperplasia and conjunctival squamous cell carcinoma with an MC-409 was highly efficient [41].

During our follow-up, we found a slight increase in the degree of dry eyes (NIKTMH decreased from 0.210 ± 0.054 mm to 0.208 ± 0.045 mm, TBUT decreased from 6.191 ± 2.811 s to 5.815 ± 2.802 s), which may be because of the injury of the corneal subbasal nerves caused by the corneal ablation, which has been reported in many studies to be associated with dry eye disease [31,42]. One complication we observed was an exacerbation of postoperative astigmatism (from 1.5 D to 8.3 D) due to intraoperative manipulation problems, which recovered 6 months later. We speculated that the cause was high local energy caused by delayed flushing during the operation, which should serve as a reminder and precaution for future doctors in such an operation. Our work has shown that the low-temperature plasma technique can control the ablation depth well and generally does not cause abnormal corneal astigmatism under normal operation.

Three patients (8.6%) in our study suffered recurrences. We analyzed the three cases to find out the possible causes and prevention plans. Case 1. A patient who had a cornea trauma for the second time from finger scratches experienced a recurrence of RCE 10 months after the ablation. The area of the second corneal erosion was distinct from the position of the first. As a result, we advise patients to prevent ocular trauma after ablation. Case 2. A patient with a history of severe EBMD had an RCE recurrence 3 months after the ablation. Since the second corneal erosion occurred in a different location than the first, we believe that it was a new RCE rather than a recurrence in the original RCE area. Additionally, we suggest clinicians to expand the ablation area for patients with severe EBMD to prevent the potential erosion of other areas of the cornea. Case 3. A patient with a history of a rubber band trauma and had an RCE recurrence one year after the ablation. The postoperative IVCM revealed a significant number of inflammatory cells in the cornea, and the recurrence area was positioned in the same location as before the procedure. We believe that the partial lesion areas were not adequately treated by the ablation procedure. We look forward to a new design in the future of the treatment head which can track and record the ablation area in order to visually cover the whole ablation range and thoroughly treat the lesion region. In the meanwhile, we suggest the importance of clinicians controlling their patients’ postoperative inflammation to help prevent the RCE from relapsing.

We also observed a significant increase in the layer of reflective particles in the corneal epithelium of patients with RCEs (Figure 3A,B). These highly reflective particles persisted after ablation. We tried to quantify the changes in these particles, but we could not because of their variable sizes. It has been suggested that these highly reflective particles are mucins, which may be related to injury repair [43]. Furthermore, Szczotka-Flynn et al., reported that mucin has antibacterial properties [44]. The specific mechanism of RCEs’ pathogenesis and highly reflective particles needs to be further explored by pathological sections and electron microscopy.

At the same time, there are some shortcomings in our study, such as the lack of a control group, limited numbers, the follow-up time not being long enough, and so forth. It is hoped that more studies will be conducted in the future to observe the efficacy of low-temperature plasma surgery for the treatment of recurrent corneal erosions. In the future, we will conduct more research on RCEs. For example, we will collect RCE patients’ tears and ocular surface tissues to find out how inflammatory factors are expressed after plasma ablation. The mechanisms of actions of the corneal tissues and macro- and micro-molecular structures using this technique should be explored further.

In conclusion, our study had shown that low-temperature plasma ablation is a safe and effective technique for the treatment of recurrent corneal erosions. Low-temperature plasma ablation is worthy of application and promotion in ophthalmic diseases.

## Figures and Tables

**Table 1:** Baseline characters of patients of RCEs.

	Patients of RCEs
Number of eyes	35
Age (years)	48.9 ± 10.9 (25–67)
Sex	16 males, 19 females
Duration (months)	9.7 ± 5.7 (3–24)
Pain score	7.7 ± 2.4 (3–10)
Follow-up (months)	12.4 ± 6.1 (6–29)
Aetiology	
Trauma	16 (45.7%)
Others	19 (54.3%)

Results are expressed as mean ± SD, RCEs, recurrent corneal erosions.

**Table 2:** Major postoperative outcome indicators.

	Preoperation	Postoperation	*p* Value
Intraocular pressure	15.5 ± 3.3	16.9 ± 3.3	0.090
Pain score	7.7 ± 2.4	1.1 ± 1.8	<0.001
Corneal astigmatism (D)	1.5 ± 1.6	0.9 ± 1.1	0.175
Corneal thickness (mm)	562.6 ± 42.2	549.6 ± 26.9	0.031
NIKTMH (mm)	0.210 ± 0.054	0.208 ± 0.045	0.001
TBUT (s)	6.191 ± 2.811	5.815 ± 2.802	<0.001
CNFL (mm/mm^2^)	10.106 ± 4.657	6.928 ± 6.620	0.028

Results are expressed as mean ±SD. NIKTMH, non-invasive keratograph tear meniscus height; TBUT, tear film breakup time; CNFL, corneal nerve fiber length.

## Data Availability

The original contributions presented in the study are included in the article/Supplementary Material; further enquiries can be directed to the corresponding author.

## Supplementary Materials

The following supporting information can be downloaded at: https://www.mdpi.com/article/10.3390/jcm11216280/s1, Video S1: An example of surgical procedures for low-temperature plasma ablation.
